# Harnessing the Potential of CRISPR/Cas in Atherosclerosis: Disease Modeling and Therapeutic Applications

**DOI:** 10.3390/ijms22168422

**Published:** 2021-08-05

**Authors:** Wei Sheng Siew, Yin Quan Tang, Chee Kei Kong, Bey-Hing Goh, Serena Zacchigna, Kamal Dua, Dinesh Kumar Chellappan, Acharaporn Duangjai, Surasak Saokaew, Pochamana Phisalprapa, Wei Hsum Yap

**Affiliations:** 1 School of Biosciences, Taylor’s University, Subang Jaya 47500, Malaysia; siewweisheng@sd.taylors.edu.my (W.S.S.); yinquan.tang@taylors.edu.my (Y.Q.T.); 2 Centre for Drug Discovery and Molecular Pharmacology (CDDMP), Faculty of Health and Medical Sciences (FHMS), Taylor’s University, Subang Jaya 47500, Malaysia; 3 Department of Primary Care Medicine, Faculty of Medicine, University of Malaya, Kuala Lumpur 50603, Malaysia; cheekei92@gmail.com; 4 Biofunctional Molecule Exploratory (BMEX) Research Group, School of Pharmacy, Monash University Malaysia, Bandar Sunway 47500, Malaysia; goh.bey.hing@monash.edu; 5 College of Pharmaceutical Sciences, Zhejiang University, 866 Yuhangtang Road, Hangzhou 310058, China; 6 Centre for Translational Cardiology, Department of Medicine, Surgery and Health Sciences and Cardiovascular Department, Azienda Sanitaria Universitaria Giuliano Isontina, Strada di Fiume 447, 34149 Trieste, Italy; Serena.Zacchigna@icgeb.org; 7 International Center for Genetic Engineering and Biotechnology (ICGEB), 34149 Trieste, Italy; 8 Discipline of Pharmacy, Graduate School of Health, University of Technology Sydney, Ultimo, NSW 2007, Australia; kamal.dua@uts.edu.au; 9 Australian Research Centre in Complementary and Integrative Medicine, Faculty of Health, University of Technology Sydney, Ultimo, NSW 2007, Australia; 10 Department of Life Sciences, School of Pharmacy, International Medical University (IMU), Bukit Jalil 57000, Malaysia; Dinesh_Kumar@imu.edu.my; 11 Unit of Excellence in Research and Product Development of Coffee, Division of Physiology, School of Medical Sciences, University of Phayao, Phayao 56000, Thailand; achara.phso@gmail.com (A.D.); saokaew@gmail.com (S.S.); 12 Center of Health Outcomes Research and Therapeutic Safety (Cohorts), School of Pharmaceutical Sciences, University of Phayao, Phayao 56000, Thailand; 13 Unit of Excellence on Clinical Outcomes Research and IntegratioN (UNICORN), School of Pharmaceutical Sciences, University of Phayao, Phayao 56000, Thailand; 14 Unit of Excellence on Herbal Medicine, School of Pharmaceutical Sciences, University of Phayao, Phayao 56000, Thailand; 15 Department of Pharmaceutical Care, Division of Pharmacy Practice, School of Pharmaceutical Sciences, University of Phayao, Phayao 56000, Thailand; 16 Department of Medicine, Division of Ambulatory Medicine, Faculty of Medicine Siriraj Hospital, Mahidol University, Bangkok 10700, Thailand

**Keywords:** CRISPR/Cas9, atherosclerosis, gene editing, gene therapy

## Abstract

Atherosclerosis represents one of the major causes of death globally. The high mortality rates and limitations of current therapeutic modalities have urged researchers to explore potential alternative therapies. The clustered regularly interspaced short palindromic repeats-associated protein 9 (CRISPR/Cas9) system is commonly deployed for investigating the genetic aspects of Atherosclerosis. Besides, advances in CRISPR/Cas system has led to extensive options for researchers to study the pathogenesis of this disease. The recent discovery of Cas9 variants, such as dCas9, Cas9n, and xCas9 have been established for various applications, including single base editing, regulation of gene expression, live-cell imaging, epigenetic modification, and genome landscaping. Meanwhile, other Cas proteins, such as Cas12 and Cas13, are gaining popularity for their applications in nucleic acid detection and single-base DNA/RNA modifications. To date, many studies have utilized the CRISPR/Cas9 system to generate disease models of atherosclerosis and identify potential molecular targets that are associated with atherosclerosis. These studies provided proof-of-concept evidence which have established the feasibility of implementing the CRISPR/Cas system in correcting disease-causing alleles. The CRISPR/Cas system holds great potential to be developed as a targeted treatment for patients who are suffering from atherosclerosis. This review highlights the advances in CRISPR/Cas systems and their applications in establishing pathogenetic and therapeutic role of specific genes in atherosclerosis.

## 1. Introduction

Cardiovascular diseases (CVDs) refer to a group of disorders that affect the heart and blood vessels, including hypertension, coronary heart disease, heart failure, rheumatic heart disease, congenital heart disease and cardiomyopathies, cerebrovascular disease, and peripheral vascular disease [1]. Atherosclerosis represents one of the main underlying causes of CVD, characterized by the presence of fibro-fatty lesions in the artery wall due to lifelong exposure to elevated low-density lipoprotein (LDL) cholesterol [2]. Lipid-lowering drugs are the primary therapeutic strategy for managing atherosclerosis. A drug such as statin helps in lowering LDL cholesterol and can be prescribed as a primary [3] and secondary prevention drug [4] for atherosclerosis treatment. Statins inhibit 3-hydroxy-3-methylglutaryl-CoA reductase (HMG-CoAR), a key enzyme which is involved in the synthesis of cholesterol. Non-statin lipid-lowering drugs, such as bile-acid sequestrants (e.g., Ezetimibe), are alternative options which inhibit the absorption of cholesterol into enterocytes of small intestine and reducing the LDL cholesterol levels. In combination therapies, statin can be combined with ezetimibe, and the treatment was shown to provide additional 15–20% reduction in LDL cholesterol levels [5]. On the other hand, there is an increasing focus on the proprotein convertase subtilisin/kexin type 9 (PCSK9) enzyme, which plays a key role in plasma cholesterol metabolism [6]. PCSK9 inhibitors such as evolocumab [7,8,9], alirocumab [10] that target PCSK9 enzyme have been tested in clinical trials and showed efficacy in lowering LDL cholesterol levels.

Considering that atherosclerosis constitutes a long pre-clinical phase, early detection of atherosclerosis may allow identification of individuals at risk for developing atherosclerotic clinical events and provides an opportunity for prevention. Both subclinical and clinical atherosclerosis has known genetic components, and novel causal therapeutic targets are being identified in several genetic studies. For instance, two prominent loci, *SERPINA1* and *AQP9*, were identified as potential candidate genes of atherosclerosis in a multi-phenotype genome-wide association study (GWAS) [11]. *SERPINA1* gene encodes for alpha 1-antitrypsin (A1AT), a protease inhibitor that enhances the degradation of LDL [12]. On the other hand, the *AQP9* gene coordinates the transport of glycerol in liver, and it is associated with reducing lipid accumulation in hepatocytes [13]. Besides, a genome-wide interaction study (GWIS) between genetic and environmental exposures uncovered several novel genetic variants in *FCAMR* (Fc fragment of IgA and IgM receptor)*-PIGR* (polymeric immunoglobulin receptor) locus that are associated with coronary atherosclerosis in individuals who are chronically exposed to traffic air pollution [14]. Another GWIS on gene-smoking interactions identified two novel genetic variants (e.g., rs1192824 and rs77461169) in the regulatory region of TBC1 domain family member 8 (*TBC1D8*) gene that affect carotid intima-media thickness and thus, increased consequent risk for atherosclerosis [15].

In line with these genetics and genomics studies, nucleic acid-based cardiovascular therapies are developing rapidly and have shown significant progress in the safety and efficacy for atherosclerosis treatment. Some prominent clinical studies of RNA-targeted nucleic acid-based therapeutics utilize small interfering RNAs (siRNAs) and antisense oligonucleotides (ASOs) to inhibit the production of proteins that are involved in lipid homeostasis such as apolipoprotein B (*APOB*), *PCSK9*, angiopoietin such as 3 (*ANGPTL3*), and apolipoprotein C3 (*APOC3*). The double-stranded siRNA has the capability to target and induce cleavage of mRNAs [16]. Inclisiran is the first-in-class cholesterol lowering siRNA conjugated to triantennary N-acetylgalactosamine carbohydrates (GalNAc) which inhibits the translation of PCSK9 and reduces levels of LDL cholesterol [17]. It was approved by EU in Dec 2020 for treatment of primary hypercholesterolaemia (heterozygous familial and non-familial) or mixed dyslipidaemia. ASO, on the other hand, is a short, single-stranded oligo that prevents protein translation by binding to mRNA target [18]. Mipomersen is an FDA approved ASO drug that binds to *APOB*-encoding mRNA, which prevents the translation of APOB and reduces LDL cholesterol level [19,20,21].

The discovery of CRISPR/Cas has emerged as an effective genome editing tool due to its ease of customization, feasibility to target almost any genome regions, and high editing efficiency with multiplexing capability. Numerous experimental studies have shown that correction of single gene defect can be achieved by the use of CRISPR/Cas technology in atherosclerosis models. This genome editing tool provides compelling alternatives to current treatment options (statins and ezetimibe), which require multiple dosages during the course of the disease. It has immense potential in facilitating development of atherosclerosis disease models and nucleic acid-based cardiovascular therapy. Despite its potential, there are two major limitations associated with this technology for its clinical translation. First, low delivery efficacy of therapeutic CRISPR tools results in non-specific targeting. Second, there are possible off-target mutations which may cause unwarranted side effects. Further research in this field is essential before it can be expanded for clinical treatment and prevention of atherosclerosis.

## 2. CRISPR/Cas System: Experimental Considerations in Atherosclerosis Models

CRISPR/Cas system was first discovered in the genome of prokaryotes in 1987 [22], but its role in adaptive immunity was not known until 2007 [23]. The basic mechanism of CRISPR/Cas genome editing has been extensively discussed elsewhere [23,24,25]. Briefly, genome editing takes advantage of the CRISPR/Cas-mediated double-strand break (DSB) at desired genome sites. DSB activates either non-homologous end-joining (NHEJ) or homology-directed repair (HDR) pathway. In NHEJ, the repair pathway mediates direct re-ligation of the excised DNA, which often results in the introduction of insertions and/or deletions (indels). The introduced indels result in either frameshift mutations or in-frame insertions/deletions, generally resulting in gene “knockout”. Conversely, HDR repair requires a donor DNA template (i.e., single or double stranded DNA) with flanking homology arms for precise replacement or repair at the cleavage site [26,27,28,29]. In general, NHEJ is the dominant repair pathway, while HDR tends to occur at a lower frequency and is usually limited to proliferating cells (i.e., S phase or G2 phase cell proliferation) [30,31,32].

Among all identified types of CRISPR/Cas systems, the one derived from *Streptococcus pyogenes* (Type II) is the most commonly used and well-characterized [33,34,35], which consists of Cas9 protein and a single guide RNA (sgRNA). Meanwhile, *Streptococcus pyogenes* Cas9 (spCas9) derivatives, such as spCas9-NG and xCas9, are also being used for genome editing. Both spCas9-NG and xCas9 variants have better PAM flexibility, and the former recognizes any target site with NG without any preference for the third nucleotide, while the latter has broader PAM compatibility that allows recognition of NG, GAT, and GAA [36,37]. Some studies utilize catalytically impaired Cas9 protein, also known as dCas9 (dead Cas9). A dCas9-gRNA ribonucleoprotein (RNP) complex can bind the promoter or regulatory regions of a target gene to induce transcriptional activation or inhibition [38,39,40,41,42,43]. Both CRISPR-activation (CRISPRa) and CRISPR-interference (CRISPRi) approaches can regulate multiple gene expressions simultaneously [44,45,46].

### 2.1. Types of Cells Used in CRISPR/Cas9 Applications

CRISPR/Cas9 can be performed on various cellular sources including somatic cells, zygotes/embryos, and pluripotent stem cells [47,48]. However, the choice of cells for genome editing application may present different technical and ethical issues. Depending on the cell type and cell state, the efficiency of DNA repair mechanisms, either NHEJ or HDR, varies substantially [49]. NHEJ can happen in most cell types including actively dividing and post-mitotic cells, whereas HDR perform better in proliferating cells [50].

Specific cell types, such as liver Kupffer cells (KCs) or liver resident macrophages, were used in the investigation of iron metabolism and atherosclerosis development. It was shown that KCs play a central role in transferring LDL-derived cholesterol to hepatocytes via ATP binding cassette subfamily A member 1 (*ABCA1*) in the presence of iron [51]. Human liver cell line Huh7, on the other hand, was used to investigate coronary artery disease (CAD) risk and atherosclerosis associated with increased milk fat globule EGF and factor V/VIII domain containing (*MFGE8*) expression [52]. Other immortal leukemic cell lines, such as K562 and Meg-01, were used to investigate the relationship between *CD36* expression and the risk of thrombo-embolism [53]. Meg-01 cell line displays phenotypic properties that resemble megakaryocytes and produces functional platelets, which is suitable for studying platelet functions [54], whereas K562 cell line possesses myelogenous origin which allowed high transfection efficiency and comparable expression profile with megakaryocytes [53].

Primary cells such as mesenchymal stem cells (MSCs) are capable of self-renewal and differentiating into various cell types, and they are recognized as a promising tool with high therapeutic utility and disease modeling [55,56]. For instance, MSCs from individuals with both atherosclerosis and T2DM have been used for the evaluation on the role of NF-κB in immuno-potency, and it was shown that constitutively active nuclear factor kappa B kinase subunit beta (IKKB) reduces the immuno-potency by changing their secretome composition [57]. Recent studies have also used MSC as a model to evaluate cardioprotective effects of *LEF1* from oxidative stress conditions [58]. Besides, the human aortic endothelial cells (HAECs) are also widely used to study endothelial pathophysiology, due to its essential role in pro- and anti-thrombotic activities as well as modulating inflammatory processes [59]. HAEC was used to investigate the effects of a noncoding polymorphism involved in endothelial mechanosensing mechanisms [60].

Human pluripotent stem cells (hPSCs) which encompass both human embryonic stem cells (hESCs) and induced pluripotent stem cells (iPSCs), is an attractive option for in vitro atherosclerosis model system [61]. Both cell lines can be reprogrammed and differentiated into specific cells for functional analysis. However, the use of hESC for research purposes remain controversial due to the use of early embryos [62]. However, the scientific community argues that hESCs should not be regarded as equivalent to embryos since the isolated hESC is unable to developed into a human being [62,63]. Meanwhile, iPSCs is genetically identical to the donor which allows reaffirmation of the patient phenotype with the in vitro cellular phenotype [61]. For instance, iPSC is used to generate vascular smooth muscle cells (VSMCs) to model the protective effects of arylacetamide deacetylase (AADAC) over-expression against atherosclerosis [64].

Nevertheless, the drawback of iPSC is that the comparison of iPSC lines between different individuals can be confusing due to the difference in epigenetics modifications and its capacities to differentiate into desired specialized cells [65]. Besides, iPSC is also prone to undesired genetic modifications during reprogramming [66]. Furthermore, genome editing in germ cells has been controversial and sparked considerable debate, where the argument revolves around permanent DNA modifications that can be passed on to future generations [67]. Hence, somatic cell genome editing appears to be more widely accepted for disease treatment of affected individuals without influencing the genetic makeup of future descendants [49].

### 2.2. Off-Target Effects of CRISPR/Cas

Off-target effects have been observed in many CRISPR/Cas9-mediated atherosclerosis model systems. For instance, a hypercholesterolemia and atherosclerosis mouse model developed by the AAV-based delivery of CRISPR/Cas9 (AAV-CRISPR) approach have detected 5% mutations in one of the predicted off-target sites in an intron of the syntaxin 8 (*STX8*) gene [68,69]. Undesired off-target effects can be reduced by using a sgRNA design tool, which helps in predicting the off-target sites across the genome [70,71] as well as using truncated sgRNA which composed of shorter number of nucleotides (<20 nt) to increase the specificity of CRISPR/Cas9 system [72]. The use of engineered Cas9 nucleases, such as enhanced *S. pyogenes* Cas9 (eSpCas9) [73] and Cas9-HF-1 [74], could provide higher on-target fidelity without affecting the cleavage efficiency. Recent studies have used two CRISPR/Cas9 nickases to flank the target DNA and generate DSB with increased specificity [75,76]. Meanwhile, other researchers have used an inactive fusion protein complex that comprises of two FokI-dCas9 fusion proteins that are recruited to adjacent target sites to facilitate efficient genome editing [77]. Modifying the binding sites of Cas9 nuclease also reduces the chance of off-target binding [78].

Meanwhile, it is suggested that different screening methods such as exome- and whole-genome sequencing (ES and WGS) are used to detect off-target events on a genome-wide scale. Specifically, the **G**enome-wide, **U**nbiased **I**dentification of **D**SBs Enabled by **seq**uencing (GUIDE-seq) and in vitro Cas9-**Di**gested whole-**genome seq**uencing (Digenome-seq) can detect specific DSBs in the genome. Guide-seq relies on the detection of double-stranded oligodeoxynucleotides in DSBs [79], while Digenome-seq involves in vitro digestion and profiling of all DSBs [80]. Another strategy to evaluate off-target assessment in vivo, also known as ‘verification of in vivo off-targets’ (VIVO), was developed and involves the identification of off-target sites using the **CIR**cularization for in vitro reporting of **CLE**avage effects by **seq**uencing (CIRCLE-seq) method [81], followed by confirmation through the targeted amplicon sequencing approach [82]. This strategy was shown to be robust and sensitive in detecting off-target mutations with minimal frequencies (0.13%) [82].

### 2.3. Types of Mutations

Genome editing can be performed in many ways to achieve the desired mutational outcome. For example, disruption of a particular gene of interest can be achieved by the formation of indels, which often cause frameshift mutations [83]. NHEJ pathway is the main mechanism involved for the gene deletion approach and often utilizes two different guide RNAs to create two DSBs flanking the target sequence [84,85]. The method can also be used to create exon skipping by inducing DSBs at two different intron regions flanking a targeted exon. Recently, it was demonstrated that similar results can be obtained by using single guide RNA only [85]. In another example, Madan et al. [53] has successfully deleted a 573 base pair fragment in vitro using two guide RNAs which flank a targeted genomic locus containing the CVD-associated genetic variants (rs2366739 and rs1194196). On the other hand, base editors and HDR approaches are both applicable for point mutation correction. Base editors are capable of precise nucleotide substitution without the need of donor template [86,87,88], whereas the HDR method requires donor template such as a copy of the wild-type gene that serves as a corrective template [32,89]. Base editors (e.g., cytosine deaminase) that fused to CRISPR/Cas9 has the ability to convert cytosine bases into uracil, and have been successfully used to introduce nonsense mutations in *PCSK9* gene [90].

### 2.4. Delivery of Genome Editing Components

Delivery of the CRISPR system into cellular or animal model systems can be challenging [91], and efficient delivery is necessary to minimize off-target effects [92]. CRISPR/Cas9 systems contain two main components—the Cas9 endonuclease and guide RNA. The two components can be delivered into the cells in different forms such as plasmids, mRNAs, and RNP. Plasmid-based method utilize plasmids containing expression cassettes for Cas9 and guide RNA, and the expression of the two components are controlled by the endogenous U6 promoter [93]. Besides, mRNA for Cas9 and guide RNA can be delivered into target cells simultaneously to achieve genome editing [94]. Next, the plasmid-free method emphasized on the formation of RNP complexes before being introduced into the cells for genome editing [93]. The RNP approach was found to exhibit higher editing efficiency with lower off-target effects in hard-to-transfect cells [95]. This method allows transient genome editing effect in transfected cells where the CRISPR/Cas9 components gradually cleared from cells over time [49].

Generally, ex vivo and in vitro genome editing can be performed using non-viral and viral delivery systems. Non-viral approach involves physical and chemical delivery strategies such as electroporation, transfection agents, nanoparticles, and cell-penetrating peptides, whereas viral delivery systems involve viral transduction using adeno-associated viruses (AAVs) or lentiviruses [91]. Electroporation method uses electrical currents to increase permeability of the cell membranes which allows the delivery of genome editing components into the cells. Electroporation method may be a better option against difficult-to-transfect cells. However, it is more laborious and expensive [96]. On the other hand, chemical methods involving the use of positively charged lipid-based nanoparticles encapsulate negatively charged nucleic acids and facilitate the delivery across the cell membranes of the targeted cells [97]. Similarly, non-lipid polymeric reagents (e.g., polyethylenimine and poly-L-lysine) share the same principle by mediating the encapsulation of CRISPR/Cas9 and allows the positively charged complexes to enter the cells via endocytosis [98].

Alternatively, viral systems offer higher genome editing efficiency in vitro/in vivo and provide the advantage of natural tropism for specific cell types, along with long-term transgene expression [49,91]. To date, the AAV viral delivery systems are frequently used for gene transduction due to its non-pathogenicity, mild immunogenicity, serotype specificity, and ability to infect proliferating and non-proliferating cells indiscriminately [99]. AAV-packaging plasmids such as adenoviral helper plasmid pAdDeltaF6 (PL-F-PVADF6) and AAV8 packaging vector pAAV2/8 (PL-T-PV0007) were co-transfected with the AAV-CRISPR plasmid into HEK293T to produce high viral titer, before intraperitoneally injected into mice. Besides, lentivirus vector is also widely used. For instance, sgRNA-Cas9-expressing lentiviruses were produced from co-transfection of gRNA-integrated Cas9-producing lentiviral plasmid (e.g., pLentiCRISPR v2) and lentiviral packaging plasmid (e.g., pMDLg/pRRE, pRSV-REV, and pVSV-G), where the former is capable of expressing CRISPR/Cas9 components upon expression, and the latter is involved in the packaging of lentivirus [57]. In addition, lentivirus has high infection efficiency, which can be a better option to transfect hard-to-transfect or non-dividing cells [100].

## 3. Therapeutic Potential of CRISPR/Cas9 System for Atherosclerosis Treatment

Development of nucleic acid-based approaches has shown promising results, the CRISPR/Cas9 gene editing technique, on the other hand, have been explored as a novel therapeutic approach for atherosclerosis. Inactivation of gene targets such as *PCSK9* [101], *APOC3* [102], and *ANGPTL3* [103] have shown to be athero-protective. For instance, CRISPR-mediated inhibition of *PCSK9* showed reduced serum PCSK9 levels and lowered plasma cholesterol by 30–40% in mice [104]. In 2016, Wang et al. [105] used the same approach on the chimeric liver-humanized mice bearing human hepatocytes and demonstrated reduced human PCSK9 levels. *PCSK9* gene modification through adenoviral delivery of CRISPR/Cas9 showed high on-target mutagenesis (close to 50%) and relatively low off-target effects [105]. Besides, robust editing of *PCSK9* (more than 40%) in murine can be achieved using a *Staphylococcus aureus* Cas9 (SaCas9) nuclease with more restrictive PAM that can reduce the probability of off-target mutagenesis [106]. Furthermore, Thakore et al. [107] demonstrated gene silencing of *PCSK9* though systemic administration of SaCas9-based repressor (dSaCas9^KRAB^) that is compatible with AAV delivery. Specific base editing was also successfully achieved by using a base editor 3 (BE3), which comprises of a CRISPR/Cas9 that is fused to a cytosine deaminase domain, and the resulting gene-edited mice showed more than 50% reduction of plasma PCSK9 protein levels and approximately 30% reduction of cholesterol levels without detectable off-target mutagenesis [108]. Moreover, following the subsequent success of in utero gene editing of *PCSK9* with positive results in murine models, gene editing before birth was made possible [109]. Hence, the idea of ‘one shot’ treatment from the elimination of liver *PCSK9* [110] in humans is appealing and seemingly possible to treat dyslipidemias. Meanwhile, *ANGPTL3*, is another new promising candidate that influences human lipoprotein metabolism by inhibiting lipoprotein lipase (LPL) [111] and endothelial lipase [112]. *ANGPTL3* gene silencing in the mouse model successfully lowered plasma cholesterols (e.g., triglyceride, HDL and LDL cholesterols) [113]. Lower triglyceride and cholesterol levels were also obtained by using CRISPR/Cas9 to mediate base editing of *ANGPTL3*, which introduced loss-of-function mutations of *ANGLTL3* in the transgenic mice model, highlighting the immense potential and feasibility of CRISPR/Cas9 technologies in gene therapy [90].

## 4. Applications of CRISPR/Cas in Atherosclerosis Models

### 4.1. In Vitro Disease Modeling

CRISPR/Cas9 tool has been widely used as a means of generating various in vitro disease models. In this section, studies that utilized CRISPR/Cas9 technology for modeling atherosclerosis are reviewed and summarized in Table 1. For instance, microsomal triglyceride transfer protein (*MTTP*)-R46G mutation has been successfully modeled in cardiomyocytes derived from iPSCs by electroporation of CRISPR/Cas9 components, which resulted in the inhibition of APOB protein expression, intracellular lipid accumulation and cell death [114]. Besides, restoration of the low density lipoprotein receptor (*LDLR*)-mediated endocytosis function as well as normalization of cholesterol metabolism have been achieved in iPSCs, by repairing the *LDLR* gene deletion with CRISPR/Cas9 [115]. Mechanistic insights of lipase A (*LIPA*) role in human macrophages were studied in human iPSC-derived macrophage model where the loss-of-function of *LIPA* exhibited reduced lysosomal acid lipase (LAL) enzymatic activity and cholesterol efflux capacity. On the other hand, iPSC-differentiated VSMCs derived from type 2 diabetes mellitus (T2DM) patients were used to investigate the protective role of *AADAC* gene [64]. CRISPR/Cas9-mediated generation of *AADAC*-knockout (KO) in T2DM patient-derived iPSC were differentiated into VSMCs. Overexpression of *AADAC* significantly diminished amount of lipid droplets in VSMCs, and amelioration of atherosclerotic lesions. Meanwhile, C-X-C motif chemokine receptor 4 (*CXCR4*)-deficient human platelets derived from iPSCs was used to investigate the functional aspects of the *CXCR4*-KO platelets [116]. Interaction between C-X-C motif chemokine ligand 14 (CXCL14) and CXCR4 was found to promote monocyte and platelet migration, and it is involved in thrombus formation, whereas *CXCR4* deficient in platelets interrupts the interaction, offering a novel therapeutic strategy for atherosclerosis treatment.

On the other hand, CRISPR/Cas9 genome editing have helped to establish an association between phosphatase and actin regulator 1 (*PHACTR1*) intronic SNPs and the locus of myocyte enhancer factor 2 (*MEF2*) binding site, suggesting the involvement of an unknown mechanisms that influence CAD/MI (coronary artery disease/myocardial infarction) risk in the vascular endothelium. CRISPR/Cas9 mediated deletion of phospholipid phosphatase 3 (*PLPP3*) gene in human aortic endothelial cells (HAECs) genome, on the other hand, demonstrated the protective role of a non-coding SNP (rs17114036) that confers increased endothelial enhancer activity, and promoted endothelial quiescence [60]. Furthermore, CRISPR/Cas9-mediated overexpression of HECT domain E3 ubiquitin protein ligase 1 (*HECTD1*) in human umbilical vein endothelial cell (HUVEC) I/R (ischaemia/reperfusion) model exhibited reduced cell viability and increased cell apoptosis and migration, providing novel insights into the relationship between *HECTD1* expression and I/R induced endothelial cell dysfunction [117]. Meanwhile, mesenchymal stem cells (MSCs) derived from patients with type-2 diabetes mellitus (T2DM) and atherosclerosis were used to investigate the effect of IKKB modulation on immuno-potency of MSCs [57]. Selective IKKB knockdown with CRIPSR/Cas9 technology in the patient-derived MSCs demonstrated reduced pro-inflammatory secretome from the deactivation of inflammatory effector (e.g., IKKB and NF-κB), which, in turn, increased survival and immuno-potency in atherosclerosis- and T2DM-patient MSCs. Meanwhile, CRISPR/Cas9 edited human umbilical cord blood-derived mesenchymal stem cells (hUCB-MSCs) that stably express *LEF1* showed significant improvement in overall survival in transplanted rats. This study has successfully demonstrated the cardio-protect effect of *LEF1* gene and potential for combining the stem cell-based therapy with genome editing technique as a therapeutic strategy for treating cardiovascular diseases [58].

Both SREBF chaperone (*SCAP*) and alanine--glyoxylate aminotransferase 2 (*AGXT2*) genes are responsible for cholesterol metabolism and nitric oxide (NO) production, and they are associated with premature myocardial infarction (MI) [118]. *SCAP* gene variant (c.3035C > T) and *AGXT2* gene variant (c.1103C > T) can be introduced into H293T and EA.Hy926 cell lines with the help of CRISPR/Cas9 technology. These edited cell lines exhibit disrupted cholesterol metabolism and reduced NO production [118,119,120]. CRISPR/Cas9-mediated deletion of *CARMA* in Huh7 demonstrated increased *MFGE8* expression. This study highlighted the roles of *CARMA/MFGE8* that may be linked to cell proliferation and/or improved survival. Meanwhile, CRISPR/Cas9 have also been used to assess the role of genomic locus that is linked to *CD36* transcription in K562 and Meg-01 cells. The research showed genomic locus variant that regulates expression of *CD36*, which, in turn, affects platelet function in these cell lines.

### 4.2. In Vivo Disease Modeling

CRISPR/Cas9 technology has made powerful breakthroughs in generating animal disease models (Table 2). Among all rodents, mice are considered organism of choice to study lipid metabolisms that contribute to diseases including atherosclerosis, due to their high genetic similarity [123]. For example, *PCSK9* gene knockout in mice, through the nanocarrier-delivered CRISPR/Cas9 system [124], and adenoviral transfection of CRISPR/Cas9 components [104] showed reduced plasma LDL cholesterol. Besides, CRISPR/Cas9 can be used to introduce human *PCSK9* gene into hypercholesterolemic mice to generate humanized animal models, as well as to introduce mutations in the human *PCSK9* gene with base editors (e.g., BE3) [125]. Remarkably, increased plasma cholesterol levels were observed in *PCSK9*-knock-in (KI) mice, whereas CRISPR/Cas9-mediated *PCSK9* knockout with BE3 reduced the plasma levels of PCSK9 and total cholesterol in the humanized mice. *LDLR* is another gene known to have direct effects on cholesterol metabolism. Somatic deletion of *LDLR* in mice can be performed using AAV-CRISPR/Cas9 system to generate atherosclerotic mice models [126]. A separate study, which induced nonsense point mutation (E208X) in *LDLR* gene using a similar approach in mice, showed severe atherosclerotic phenotypes [127]. The mutation was shown to be corrected by subcutaneous injection of AAV-CRISPR/Cas9 system which demonstrated partial restoration of *LDLR* expression, with reduced total cholesterol, triglyceride, and LDL cholesterol levels [127]. Double gene knockout has also been successfully performed in mice models where the *LDLR/APOE* deficiency exhibits prominent atherosclerosis phenotypes [128,129].

Down-regulation of other gene targets such as *ANGPTL8*, *FMO3*, and *STXBP5* were also modeled in mice and displayed impaired lipogenesis with reduced body weight and plasma triglyceride levels [130], reduced thrombosis potential and atherosclerosis [131], and lowered plasma von Willebrand factor (VWF) levels with reduced thrombosis [132]. Besides, conditional Geranylgeranyl transferase-I (GGTase-I) knockout mice was generated to study the GGTase-I enzyme, which is involved in the mediation of post-translational modification (e.g., geranylgeranylation) of small GTPase, Rac1 [133]. GGTase-I produced from the expression of protein geranylgeranyltransferase type I subunit beta (*PGGT1B*) gene is associated to diabetes-accelerated atherosclerosis, and removal of *PGGT1B* gene in mice shown attenuated phenotype of diabetes-accelerated atherosclerosis with possible involvement of several mechanisms that inhibit VSMC proliferation [133].

Hamster, on the other hand, serves as a suitable choice for hyperlipidemia translational research due to several advantages which include its capability of producing cholesteryl ester transfer protein, feasibility for intestine-only *APOB* editing, and low hepatic cholesterol synthesis properties [134]. Hybrid strain of golden Syrian hamsters was found to respond to high-cholesterol diets and greater susceptibility to atherosclerosis, which makes it an excellent choice for generating atherosclerosis models with prominent cardiovascular pathophysiology manifestation [135]. Besides, the *LDLR*-KO hamsters can be induced by microinjecting CRISPR/Cas9 components into fertilized eggs for the development of hypercholesterolemia and hyperlipidemia model [134]. Meanwhile, *LCAT*-deficient hamsters can be generated using similar approach and the resulting adult hamsters exhibited pro-atherogenic dyslipidemia [136]. Novel homozygous apolipoprotein C2 (*APOC2*)-ablated golden Syrian hamster that exhibits severe hypertriglyceridemia was established with CRISPR/Cas9, and the genetically modified hamster is useful to study the APOC2 function and its effect on lipid and glucose homeostasis [137]. Recently, Guo et al. [138] showed anti-atherogenic effects in *APOC3*-KO golden Syrian hamster, suggesting that CRISPR-mediated knockout of *APOC3* in human may be a potential therapeutic approach in alleviating atherosclerosis.

Zebrafish has been regarded as a useful model to study cardiovascular diseases, serving as an excellent tool for rapid, simple and low-cost system for drug screening [139]. Thrombocytes in zebrafish are homologous to mammalian platelets, thus zebrafish become an excellent model to study thrombosis in mammals [140,141]. Specific heart development protein with EGF such as domains 1 (*HEG1*) knockout in zebrafish line with CRISPR/Cas9 showed severe cardiovascular malformations [142]. On top of that, thrombosis phenotype was observed with venous thrombosis and slow blood flow, which were similar to human heart failure. The *HEG1*-specific knockout zebrafish line was established as a new model of dilated cardiomyopathy (DCM) and thrombosis and verified to be appropriate for drug screening.

Disease modeling involving rabbits are frequently used to study atherosclerosis due to their similar lipoprotein metabolism with humans [143,144]. Rabbits are known to have abundant plasma cholesteryl ester transfer protein (*CETP*), which is advantageous for studying CETP and its relationship with atherosclerosis [145,146]. Interestingly, genetic ablation of *CETP* gene in the rabbit model demonstrated athero-protective effects, suggesting a potential therapeutic target for atherosclerosis treatment [146]. Meanwhile, the Watanabe heritable hyperlipidemic (WHHL) rabbits are often used as a human familial hypercholesterolemic model due to its characteristic high LDL levels [147]. Following the discovery of CRISPR/Cas9, knockout rabbit models are made possible with the capability to target different gene of interests [148]. The first reported CRISPR-KO rabbits for the investigation of lipid metabolism was demonstrated by Niimi et al. [149]. In addition, CRISPR/Cas9-mediated knockouts of *LDLR* and *LDLR/APOE* double-knockout in rabbits successfully demonstrated reduced high-density lipoprotein (HDL) cholesterol levels, severe dyslipidemia and atherosclerotic lesions in the rabbits aorta [143,150]. However, recent studies highlighted that rabbits lack of calponin 2, an actin-associated cytoskeletal protein involved in the pathogenesis of diseases including atherosclerosis, and the use of rabbit model for replicating human diseases require cautious consideration [144]. On the other hand, two different studies have successfully produced *APOE^−/−^* and *LDLR*-KO pigs by introducing indels in primary pig fetal fibroblasts (PFFs) and porcine embryonic fibroblasts (PEFs), respectively. The edited cells were subsequently used as nuclear donors for the reconstruction of pig embryos using the somatic cell nuclear transfer (SCNT) method [151,152]. These studies have demonstrated the feasibility of CRISPR/Cas9 across different animal models of dyslipidemia and atherosclerosis and shed light to the underlying molecular mechanisms, paving the way for development of novel therapeutics and treatment possibilities.

## 5. Clinical Application of Genome Editing in Atherosclerosis Patients

The use of genome editing as a form of therapeutic option constitutes an exciting research area. The first gene therapy clinical trial dated back in September 1990 used genetically modified T-cells of a patient suffering from adenosine deaminase deficiency to restore the gene function [154]. Since then, the trial acted as a foundation for all subsequent nucleic acid-based therapies developed. Ex vivo therapy using CRISPR technology, has advantages over the in vivo approach in the aspect of technical feasibility and safety as gene editing and generation of respective edited cells are performed under controlled environment. For example, hUCB-MSCs has been used as a potential development of stem cell-based therapy of ischemic heart diseases by generating the CRISPR edited hUCB-MSCs *ex vivo*, before transplanting into the infarction region of mice [58]. The experiment showed overall improvement of the survival in mice. Several clinical trials involving CRISPR ex vivo genome editing were already initiated, for instance, to treat leukemia/lymphoma patients (clinical-trial.gov: NCT04037566), sickle cell disease patient (clinical-trial.gov: NCT03745287), and others. [155]. On the other hand, in vivo genomic editing strategy could directly edit the target cells. Systemic delivery of CRISPR/Cas9 components were shown to restore the dystrophin (*DMD*) reading frame and improve overall heart functionality in the mouse model [156]. Breakthrough was made by the generation of human disease modeling using human pluripotent stem cell (PSC) lines, where the SNP of interest was successfully introduced to myosin heavy chain 7 (*MYH7*) gene [157]. The study highlighted the capability of CRISPR/Cas9 in producing isogenic cell lines/models, which would be useful for evaluating new therapies and paving the way for gene-based therapeutics. Furthermore, CRISPRi was used in devising a novel therapeutic strategy for patient with long-QT syndrome [158]. The strategy involved the use of dCas9 fused with a Krüppel associated box (KRAB) suppressor to target and suppress the mutant gene in iPSC-CM derived from a patient, which demonstrated functional rescue phenotypes (e.g., normalized action potential, and Ca^2+^/CaM-dependent inactivation) after treatment. Altogether, these studies provided proof-of-concept evidence on the potential use of CRISPR/Cas9 technology in CVDs treatment. However, rigorous assessment and evaluation on the safety of use and efficacy in large trials are needed.

## 6. Conclusions

The CRISPR/Cas system is a powerful genome editing tool for manipulating the genome and investigating the pathophysiological mechanisms of atherosclerosis using in vitro and in vivo experimental models. Various CRISPR/Cas applications, such as single-base editing, epigenetic modifications, live-cell imaging, CRISPRi, and CRISPRa have been utilized in the field of cardiovascular medicine to dissect the molecular pathogenesis of atherosclerosis, and it can potentially be used as a therapeutic tool for targeting atherosclerosis-associated diseases such as hyperlipidemia and hyperglycemia. Nevertheless, challenges, including off target mutagenesis, delivery efficiency of genome editing tools, lower success rate of HDR in non-proliferating cells, and the potential of Cas9-triggered immune responses in the human body, need to be taken into considerations during the development of CRISPR therapeutics. Despite its practicality, ethical conflicts regarding the use of CRISPR/Cas system in human subjects remains a huge barrier. Hence, future advancement of CRISPR/Cas system is essential to achieving better efficacy and long-term safety in CRISPR-based therapy.

## Figures and Tables

**Table 1:** CRISPR/Cas9 genome editing in atherosclerosis models (in vitro).

Type of Cells	Targeted Genes	Type of Mutation	Delivery Strategy	CRISPR Components	Model Phenotype	Reference
iPSCs	*LDLR*	Knock-in	Electroporation	Cas9n, sgRNAs plasmids, and ssODN	Restored LDLR-mediated endocytosis and showed normalized cholesterol metabolism in hepatocytes.	[115]
iPSCs, HEK293T, Neuro-2a	*PCSK9*	Knockout	No information	BE3-encoding plasmid and sgRNA	Reduced plasma PCSK9 and cholesterol levels.	[108]
iPSC-derived cardiomyocytes, human hepatocytes	*MTTP*	Knockout and knock-in	Electroporation	sgRNA-Cas9 expressing plasmid and ssODNs	*MTTP*-knockout human hepatocytes and cardiomyocytes exhibit loss of APOB secretion, accumulated intracellular lipid, and increased cell death; the adverse phenotypes were reversed in corrected hepatocytes and cardiomyocytes.	[114]
iPSCs-derived platelets	*CXCR4*	Knockout	Electroporation	sgRNA-Cas9 expressing plasmid	Deficiency of *CXCR4* in iPSC-derived platelets disrupted CXCR4 interaction with CXCL14 and inhibit platelet migration.	[116]
iPSCs-derived macrophages	*LIPA*	Knockout	Electroporation	Cas9-GFP and sgRNA expressing plasmids	*LIPA*-KO macrophages showed low LAL enzymatic activity, suggesting deficiency in lysosomal cholesteryl ester hydrolysis.	[121]
iPSC-derived VSMCs	*AADAC*	Knockout	Electroporation	Cas9-GFP and sgRNA expressing plasmids	Reduced expression of *AADAC* resulted in significant increase of lipid droplets in VSMCs; overexpression of *AADAC* in vitro and in vivo diminished lipid droplets and ameliorated atherosclerotic lesions.	[64]
Huh7	*CARMA*	Knockout	Lentiviral transduction	Lenti-iCas9-neo, psPAX2 and pMD2.G plasmids	*CARMA* (CAD Associated Region between *MFGE8* and *ABHD2*) deletion increases *MFGE8* expression; it is associated with increased proliferation of smooth muscle cells and monocytes.	[52]
K562, Meg-01	*CD36*	Knockout	Lipofectamine 2000 reagent /Nucleofection	AIO-GFP(Cas9)-sgRNA expressing plasmid	The deletion of a genomic locus variant in K562 and Meg-01 cells resulted in upregulation of *CD36* transcription and platelet activation.	[53]
HEK293T, EA.Hy926	*SCAP, AGXT2*	Knockout	Lipofectamine 2000 reagent	sgRNA-Cas9 expressing plasmid	The *SCAP* variant impaired activation of cholesterol synthesis-related genes and *AGXT2* variant leads to down-regulation of nitric oxide production. The two CRISPR-induced variants are associated with premature myocardial infarction.	[118]
Patient-derived MSCs	*IKKB*	Knockdown	Lentiviral transduction	pLentiCRISPR v2, pMDLg/pRRE, pRSV-REV and pVSV-G plasmids	Reduced production of pro-inflammatory secretome, improved survival and immuno-potency in patient-derived MSCs.	[57]
Human umbillical cord blood-derived-MSCs	*LEF1*	Knock-in	Lipofectamine 3000	*LEF1*-overexpressing plasmid	*LEF1*-overexpressing hUCB-MSCs showed activation of canonical Wnt/β-catenin signaling and cyclin D1 expression; reduced MI region and fibrosis.	[58]
hESCs	*PHACTR1*	Knockout	Electroporation	Cas9-GFP and sgRNA plasmids	Established an association between the *PHACTR1* SNP with CAD/MI risk.	[122]
HAECs	*PLPP3*	Knockout	Lipofectamine RNAiMAX reagent	Cas9-sgRNA RNP complex	Deletion near rs17114036 location showed reduced *PLPP3* expression and promoted endothelial quiescence from unidirectional shear stress.	[60]

BE3, base editor 3; CAD, coronary artery disease; CAD/MI, coronary artery disease/myocardial infarction; Cas9n, Cas9 nickase; EA.Hy926, permanent human endothelial cell line; GFP, green fluorescent protein; HAECs, human aortic epithelial cells; HEK293T, human embryonic kidney 293 cell line; Huh7, hepatocellular carcinoma Huh7 cell line; hESCs, human embryonic stem cells; iPSC, induced pluripotent stem cells; KO, knockout; K562, human erythroleukemic cell line; LAL, lysosomal acid lipase; Meg-01, megakaryoblastic cell line; MI, myocardial infarction; MSCs, mesenchymal stem cells; Neuro-2a, neuro-2a neuroblastoma cell line; RNP, ribonucleoprotein; sgRNA, single guide RNA; ssODN, single-stranded oligo DNA nucleotide; VSMCs, vascular smooth muscle cells.

**Table 2:** CRISPR/Cas9 genome editing in atherosclerosis models (in vivo).

Disease Model	Targeted Genes	Type of Mutation	Delivery Strategy	CRISPR Components	Model Phenotype	Reference
Mice	*FMO3*	Knockout	Electroporation into embryonic stem cells; microinjection into mice blastocysts	Promoter-driven KO first targeting plasmid	Reduction in trimethylamine N-oxide levels, thrombosis potential in *FMO3*-KO mice.	[131]
Mice	*LDLR*, *APOE*	Double-knockout	Microinjection	sgRNAs (*APOE*- and *LDLR*-targeting), and Cas9	Development plaques, destruction of pancreatic islets, inflammatory response.	[128]
Rat	*LDLR*, *APOE*	Double-knockout	Microinjection	Cas9 mRNA and sgRNAs (*LDLR*- and *APOE*-targeting)	Severe dyslipidemia, liver steatosis, and atherosclerotic plaques in the aorta.	[129]
Mice	*LDLR*	Knockout	Adenoviral transduction; intraperitoneal injection	Plasmid 1375 (sgRNA and Cas9-encoding plasmid), pAdDeltaF6, and pAAV2/8 plasmids	Severe hypercholesterolemia and atherosclerotic lesions in the aorta.	[126]
Mice	*LDLR*	Knock-in	Adenoviral transduction; subcutaneous injection	Cas9- and sgRNA-donor-expressing AAV plasmids, and AAV8-GFP plasmid	*LDLR*-E208X point mutation was corrected in mice, and it showed partially restored *LDLR* expression, reduced total cholesterol, triglyceride, and serum LDL cholesterol.	[127]
Mice	*LDLR*	Knock-in	Tail vein injection	AAV8.TBG.mLDLR/AAV8.TBG.hLDLR	Reduced serum cholesterol level.	[153]
Mice	*PCSK9*	Knockout	Intravenous injection	GAL-LGCP encapsulated TAT-GNCs-Cas9-sgPCSK9 complex	Reduced plasma LDL cholesterol levels.	[124]
Mice	*PCSK9*	Knockout	Retro-orbital injection	BE3-sgRNA-expressing AAV	BE3 base editing of *PCSK9* in mice liver demonstrated reduced plasma PCSK9 and cholesterol levels.	[108]
Mice	*PCSK9*	Knockout	Adenoviral transduction; retro-orbital injection	Cas9-sgRNA expressing recombinant plasmid and GFP-expressing plasmid	Decreased plasma PCSK9, increased expression of hepatic LDL receptor and decreased plasma cholesterol levels.	[104]
Mice	*PCSK9*	Knockdown	Calcium phosphate precipitation; tail vein injection	dSaCas9^KRAB^ and gRNA lentiviral expression plasmids, psPAX2, and pMD2.G plasmids	Decreased plasma PCSK9 and cholesterol levels.	[107]
Mice	*PCSK9*	Knock-in and Knockout	Tail vein injection	Cas9/BE3-encoding plasmid	Development of humanized mouse model with liver-specific expression of human *PCSK9* and a human-like hypercholesterolemia phenotype.	[125]
Mice	*PGGT1B*	Knockout	Microinjection	Cas9 mRNA, sgRNA, and donor plasmids	Attenuated phenotype of diabetes-accelerated atherosclerosis in vivo which is associated to the inhibition of VSMC proliferation.	[133]
Mice	*STXBP5*	Knock-in	Microinjection	SpCas9 mRNA, SNP (437Asn)-targeting sgRNA, and ssODN	*STXBP5* SNP (rs1039084) mice model showed lower plasma VWF levels, reduced thrombosis and reduced platelet secretion compared to wild-type mice.	[132]
Hamster	*LCAT*	Knockout	Microinjection	Cas9 mRNA and sgRNAs	Pro-atherogenic dyslipidemia in *LCAT*-deficient hamster; high fat diet increases atherosclerotic lesions.	[136]
Hamster	*LDLR*	Knockout	Microinjection	sgRNA and Cas9 mRNAs	Chow diet hamsters developed hypercholesterolemia, hyperlipidemia; HCHF diet hamsters developed atherosclerotic lesions in the aorta and coronary arteries.	[134]
Rabbit	*LDLR, APOE*	Double-knockout	Microinjection	sgRNA and Cas9 mRNAs	Hyperlipidemia with prominent aortic and coronary atherosclerosis with accumulated atherosclerotic lesions consisted macrophage foam cells in rabbit model.	[150]
Rabbit	*LDLR*	Knockout	Microinjection	sgRNA and Cas9 mRNAs	*LDLR*-KO rabbit exhibited increased plasma triglycerides, LDL cholesterol, and reduced HDL cholesterol levels; prominent aortic and coronary artery atherosclerosis were observed.	[143]
Pigs	*APOE*	Knockout	Electroporation; SCNT	Cas9-sgRNA expressing plasmid	*APOE*-KO pigs on HFHC diet displayed severe hypercholesterolemia, and atherosclerotic lesions in the aorta and coronary arteries.	[151]
Pigs	*LDLR, APOE*	Double knockout	Electroporation; SCNT	Cas9 and gRNA plasmids	*APOE*- and *LDLR*-KO SCNT pigs demonstrated increased LDL cholesterol, total cholesterol and apolipoprotein B levels.	[152]
Zebrafish	*HEG1*	Knockout	Microinjection	gRNA and Cas9 protein	Mutant zebrafish model demonstrated severe cardiovascular malformations and thrombosis phenotype such as venous thrombosis and slow blood flow.	[142]

AAV, adeno-associated virus; BE3, base editor 3; GAL-LGCP, Gal-conjugated PEG–lipid/TAT–GNCs/Cas9/sgPCSK9; GFP, green fluorescent protein; GNCs, gold nanoclusters; HCHF, high-carbohydrate high-fat; HDL, high-density lipoprotein; HFHC, high-fat and high-cholesterol; KO, knockout; LDL, low-density lipoprotein; mRNA, messenger RNA; SCNT, somatic cell nuclear transfer; sgRNA, single guide RNA; SNP, single nucleotide polymorphism; SpCas9, *Streptococcus pyogenes* Cas9; ssODN, single-stranded oligo DNA nucleotide; TAT, HIV-1-transactivating transcriptor; VSMC, vascular smooth muscle cell; VWF, von Willebrand factor.

## Data Availability

Not applicable.
